# Apocynin: Molecular Aptitudes

**DOI:** 10.1155/2008/106507

**Published:** 2008-12-02

**Authors:** J. Stefanska, R. Pawliczak

**Affiliations:** Department of Immunopathology, Medical University of Lodz, 251 Pomorska Street, Building C5, 92-213 Lodz, Poland

## Abstract

Apocynin is a naturally occurring methoxy-substituted catechol, experimentally used as an inhibitor of NADPH-oxidase. It can decrease the production of superoxide (O_2_^−^) from activated neutrophils and macrophages while the ability of phagocytosis remains unaffected. The anti-inflammatory activity of apocynin has been demonstrated in a variety of cell and animal models of inflammation. Apocynin, after metabolic conversion, inhibits the assembly of NADPH-oxidase that is responsible for reactive oxygen species (ROS) production. It is, therefore, extensively used to reveal the role of this enzyme in cell and experimental models. Although some of the ROS serve as signaling molecules in the cells, excessive production is damaging and has been implicated to play an important role in the progression of many disease processes. This is why in many studies apocynin presents a promising potential treatment for some disorders; however, its utility with inflammatory diseases remains to be determined. Since its mode of action is not well defined, we tried to get a more precise insight into the mechanisms by which apocynin exerts its activity. Considering the anti-inflammatory activities of apocynin, we may conclude that this compound definitely deserves further study.

## 1. INTRODUCTION

Oxidative stress describes an imbalance between reactive oxygen species (ROS) synthesis and antioxidants. The normal production of oxidants is counteracted by several antioxidative mechanisms in the body [1, 2].

Normally, cells possess antioxidant defense systems that include ROS degrading molecules (ROS scavengers), such as uric acid, ascorbic acid, and sulfhydryl-containing molecules (e.g., glutathione), and antioxidant enzymes, such as catalase, glutathione peroxidase, and superoxide dismutases. In pathologic conditions, in which excessive production of ROS outstrips endogenous antioxidant defense, oxidative stress may irreversibly modify (oxidize) biologic macromolecules such as DNA, protein, carbohydrates, and lipids. Enhanced generation of O_2_^−^ also causes loss of NO bioavailability. O_2_^−^ and NO undergo a very fast radical-radical termination reaction to yield a secondary oxidizing species, peroxynitrite anion (ONOO^−^), and peroxynitrous acid. ROS induce apoptotic cell death in various cell types, and deregulation of apoptosis causes clinical disorders [3]. NADPH-oxidase is the enzyme responsible for ROS production, and inhibition of this enzyme represents an attractive therapeutic target for the treatment of many diseases. To counteract oxidative stress, the body produces an armory of antioxidants to defend itself, which in fact are sometimes insufficient to effectively defend the organism from ROS [2, 4]. There are a lot of substances that have been researched in order to find a way to inhibit production of ROS, and thus protect the body from diseases. Apocynin, NADPH-oxidase inhibitor, is one of such agents; however, it is considered variously in publications. Some of these were found to have potential clinical success while others have already been abandoned again, as will be discussed in this short selective review.

## 2. APOCYNIN

The apocynin (4-hydroxy-3-methoxyacetophenone, trivial names: apocynin, acetovanillone) was first described by Schmiedeberg in 1883 and was isolated from the roots of *Apocynum cannabinum* (Canadian hemp), and extracts of it were used as official remedies for dropsy and heart troubles. In 1971, apocynin was identified during activity-guided isolation of immunomodulatory constituents from the root of *Picrorhiza kurroa* (*Scrophulariaceae*), a native plant grown in the mountains of India, Nepal, Tibet, and Pakistan, well known in traditional Indian medicine (Ayurveda). Apocynin is an acetophenone with a molecular weight of 166.17 and forms needles upon crystallization from water. It possesses a faint vanilla odor and has a melting point of 115°C [4, 5].

Apocynin has been used as an efficient inhibitor of the complex NADPH-oxidase in many experimental models involving phagocytic and nonphagocytic cells [6, 7]. The mechanism of inhibition is not totally known, but involves the impairment of the translocation to the membrane of the cytosolic component p47phox of the NADPH-oxidase complex [8, 9].

**Figure 1:**
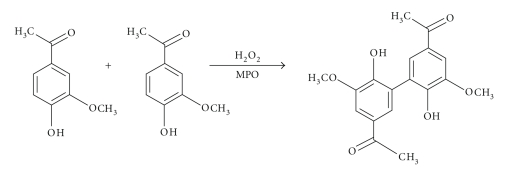
Creating active form of apocynin-dimerization.

A very important finding concerning this mechanism was the discovery that apocynin is a prodrug that is converted by peroxidase-mediated oxidation to a dimer, which has been shown to be more efficient than apocynin itself [10] (Figure 1).

Apocynin is an inhibitor of the intracellular translocation of two critical cytosolic components of the NADPH-oxidase complex present in the cell membrane [9].

The structure of NADPH-oxidase is quite complex, consisting of two membrane-bounded elements (gp91phox and p22phox), three cytosolic components (p67phox, p47phox and p40phox), and a low-molecular-weight G protein (either rac 2 or rac 1). The racs are kept inactive by binding to a guanine nucleotide dissociation inhibitor, which prevents the exchange of guanine nucleotides from the rac proteins [5]. Activation of NADPH-oxidase is associated with, and probably caused by, the migration of the cytosolic components to the cell membrane so that the complete oxidase can be assembled [11].

Apocynin is a selective inhibitor of NADPH-oxidase activity and concomitant ROS production (IC50 value: 10 *μ*M) in activated human neutrophils [12]. Interestingly, it does not seem to interfere with the PMNs other defense mechanisms, as it does not affect phagocytosis or intracellular killing [2].

**Figure 2:**
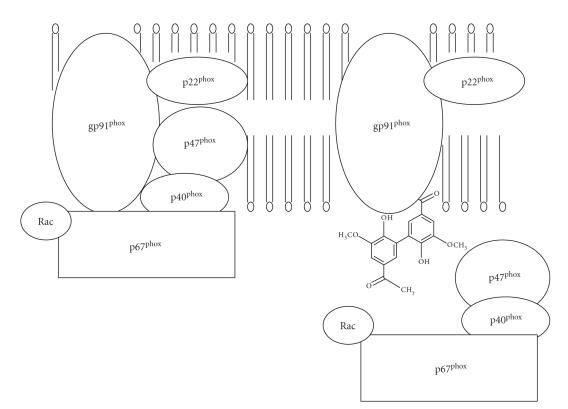
The mechanism of NADPH-oxidase inhibition by apocynin.

Apocynin prevents the translocation of p47phox to Nox2 in leukocytes, monocytes, and endothelial cells [2, 10] (Figure 2). The inhibitory action of the compound, however, occurs after a lag time only. The latter process appears to involve MPO because apocynin does not inhibit the oxidase in cells devoid or deficient of MPO [13], and agents such as zymosan that promote the release of MPO enhance the efficacy of apocynin [14].

In the original report of apocynin, as an NADPH-oxidase inhibitor, it was noted that the compound requires activation by myeloperoxidase (MPO) [12]. It is assumed that apocynin is activated by H_2_O_2_ and MPO to form an apocynin radical, which then oxidizes thiols in the NADPH-oxidase. Indeed, thiols are critical for the function of p47phox, and thiol oxidizing agents have been shown to block NADPH-oxidase activation [10, 15]. In line with this concept, it was observed that supplementation of thiol provided either as glutathione or cysteine prevents the inhibitory effect of apocynin on the NAPDH oxidase. An alternative explanation for the lag time of the inhibitory effect of apocynin was that through the step of an apocynin radical, an apocynin dimer is formed [16]. In fact, it has been suggested that this dimer only is the active inhibitory compound that may block NADPH-oxidase activity [14].

The in vitro anti-inflammatory effects of apocynin include the following:

- the reduction of neutrophil oxidative burst,
- neutrophil-mediated oxidative damage [12],
- the decreased adhesion of the monocytic cell line U937 to tumor necrosis factor (TNF) treated human umbilical vein endothelial cells [5],
- a reduction of polymorphonuclear granulocyte chemotaxis [17],
- the inhibition of peroxynitrite [18],
- the inhibition of inflammation-mediated cartilage destruction [6].

Not much is known about the kinetics of apocynin in vivo, but interesting metabolic aspects of apocynin were described by Daly et al. [19]. He showed that after a period of 20 hours upon IP administration of 120 mg/kg apocynin to rats, 80% of the apocynin was recovered unchanged in the urine of the animals. Side effects of apocynin are not known. Apocynin has very low toxicity (LD50: 9 g/kg) after oral administration in mice [20].

## 3. APPLICATION: RESPIRATORY SYSTEM AND ASTHMA

Ischemia-reperusion lung injury is a well-known clinical phenomenon characterized by increased pulmonary vascular permeability, edema, and resistance to blood flow [21, 22]. The pathogenesis of this injury appears to involve the generation of ROS, which can be detected during both ischemia and reperfusion [23, 24]. The ability of superoxide dismutase to attenuate ischemia-reperfusion lung ininjury suggests that superoxide anion production represents a critical step in the process [25, 26]. Apocynin has been shown to confer protection in animal models of arthritis [27], and in ozone and endotoxin-induced lung injury [13, 28]. Doddo et al. [21] showed that apocynin prevents the increased vascular permeability caused by ischemia and reperfusion in isolated sheep lungs. The effect of apocynin on the changes in vascular permeability after ischemia-reperfusion was dose-dependent. This suggests that apocynin is able to maintain normal endothelial albumin permeability during ischemia and reperfusion.

*P. kurroa*, as mentioned previously, has been used for ages in the treatment of asthma. Peters et al. [9] observed a statistically significant and pronounced effect of apocynin on ozone-induced bronchial hyperresponsiveness to methacholine measured 16 hours after exposure to ozone in mild asthmatics. In addition, apocynin did not prevent the decline of FEV_1_ measured directly after ozone exposure, excluding a possible scavenger effect by apocynin of ozone. These results suggest that apocynin may have a role in preventing ozone-induced exacerbations of asthma and extend the results of Kudo et al. which reported the effect of apocynin on ozone-induced airway hyperresponsiveness in guinea pigs [29, 30]. The publication showed that superoxide dismutase, a scavenger of superoxide, as well as apocynin, reduced O_3_-induced airway hyperresponsiveness. It was previously described that peroxidase activation of apocynin is a prerequisite for inhibition of NADPH-oxidase and that this activated apocynin is unlikely to work at distances such as in the airway epithelium [9].

A possible explanation for the effectiveness of apocynin in the treatment of respiratory diseases might be the fact that apocynin inhibits peroxynitrite (ONOO^−^) formation [18]. ONOO^−^ is the very reactive product of the reaction of nitric oxide (NO) and superoxide anion (^•^O_2_^−^). For many years, much attention has been paid to the effects of NO in respiratory diseases [31], but recently, the focus has been shifted toward reactive nitrogen species (RNS) in general, and to peroxynitrite in particular [32, 33]. Peroxynitrite is suggested to induce epithelial damage, mediator release, and consequently hyperresponsiveness [34]. This finding may have important clinical implications since airway inflammation, epithelial damage, and hyperresponsiveness are characteristic features in patients suffering from asthma.

## 4. APPLICATION: NEUROPROTECTIVE FEATURES

NADPH-oxidase-mediated superoxide plays an important role in the pathogenesis of brain injury and that inhibition of NADPH-oxidase by apocynin can attenuate brain injury following experimental ischemic stroke [35]. It has been reported that apocynin protects against global cerebral ischemia-/reperfusion-induced oxidative stress and injury in the gerbil hippocampus [36], and inhibiting superoxide production by NADPH-oxidase with apocynin protects blood-brain barrier constituents in ischemia-like injury in vitro [37]. Wang et al. investigated that apocynin was able to protect against brain injury in a collagenase-induced rat model of intracerebral hemorrhage (ICH). Apocynin reduced cerebral and vascular injury in experimental stroke models at doses similar to those used in the present study [38].

On the other hand, Titova et al. claim that apocynin should not be considered as a potential therapeutic agent in ICH, as ICH results in the activation of NADPH-oxidase and brain injury that cannot be countered by a clinically relevant administration of apocynin. Additionally, it is reported that apocynin had no effects not only on enhanced NADPH-oxidase activity but also on lipid peroxidation and brain water content, as well as the profound neurological dysfunction that occurred after ICH [39].

However, the results of Tang's study suggest that an increase in NADPH-oxidase activity may contribute to the enhancement of superoxide in the infarct area, and NADPH oxidase-mediated superoxide production may play a crucial role in the pathogenesis of ischemic brain injury. Apocynin has been shown to confer protection in animal models of ischemia-/reperfusion-induced lung injury. It was administrated intraperitoneally, and treatment with apocynin significantly decreased NADPH-oxidase activity and reduced the superoxide level, in addition to attenuating significantly the size of the infarct [35].

Inflammation following ischemic stroke is known to contribute to injury. Apocynin has been studied as a potential treatment in experimental stroke. Tang et al. [40] explored the effect of different doses of apocynin in a mouse model of 2-hour transient middle cerebral artery occlusion followed by 22-hour reperfusion. Apocynin, given at a dose of 2.5 mg/kg 30 minutes before reperfusion, improved neurological function, reduced infarct volume, and reduced the incidence of cerebral hemorrhage. Nevertheless, at higher doses of 3.75 and 5 mg/kg, it increased brain hemorrhage. Apocynin also tended to reduce mortality at the lower dose, but not at higher doses. This data suggest that apocynin can protect against experimental stroke, but with a narrow therapeutic window [40].

## 5. APPLICATION: ARTERIOSCLEROSIS AND HYPERTENSION

Atherosclerosis is one of the most common cardiovascular diseases in developed countries and is yet another disease in which ROS are thought to play an important role [41]. Among the main causes of the development of atherosclerosis is a high serum level of low-density cholesterol-containing lipoprotein [42].

Experiments with apocynin in endothelial cells showed similar results compared with the effects of apocynin in phagocytes. Holland et al. reported that endothelial cells, incubated with apocynin (600 *μ*M) and stimulated with the phospholipase A2 activator thrombin, showed NADPH-oxidase inhibition, resulting in a significantly impaired ROS production [43]. The same authors also state that endothelial cell incubation with apocynin markedly diminished high LDL-induced increases in cellular H_2_O_2_ concentrations [44]. Furthermore, apocynin was shown to be effective at suppressing atherogenesis in vivo in spite of highly elevated serum low density lipoprotein (LDL) levels using a rabbit model [45]. So, maybe apocynin has revealed a new strategy in the treatment of atherosclerosis, and, therefore, future treatments should also focus on NADPH-oxidase inhibition as an effective way of preventing the endothelium from the initiating events of atherosclerosis.

Apocynin is a reversible inhibitor of NAD(P)H oxidase activity that impedes assembly of the p47phox subunit with the membrane complex [46]. It has recently been proposed as a potential therapeutic agent in the treatment of atherosclerotic disease by Meyer and Schmitt [47]. Increased levels of reactive oxygen species, in particular O_2_^−^, are a major cause of endothelial dysfunction in many forms of cardiovascular disease. One of the most important sources of O_2_^−^ is NAD(P)H oxidases [48]. Hamilton et al. [49] have shown that treatment of isolated rat arteries with apocynin decreased NADH-stimulated O_2_^−^ generation and increased NO bioavailability. In addition, in human arteries and veins, NADH- and NADPH-oxidase-mediated O_2_^−^ generation was inhibited by apocynin; endothelium-dependent vasodilation was improved; and NO production from human SV endothelial cells was enhanced [49].

Nox2 (formerly known as gp91phox) expression is upregulated and is associated with elevated endothelial and adventitial NADPH-oxidase-dependent superoxide production in model of vascular remodelling, leading to endothelial nitric oxide dysfunction [50, 51].

Direct inhibition of the source of superoxide generation with apocynin may well be a useful approach to reduce the remodelling associated with the early stages of postangioplasty remodelling or atherosclerosis [47]. Local administration of an NADPH-oxidase inhibitor apocynin in vivo to the collared arteries attenuates superoxide production and prevents vascular injury associated with this remodelling [21].

Apocynin treatment of the collared arteries did not affect Nox2 mRNA expression, thus indicating that apocynin reduced superoxide generation in the collared arteries by suppressing the NADPH-oxidase activity rather than affecting its gene transcription. Apocynin has been demonstrated to reverse endothelial NO dysfunction in animals or humans subjected to oxidative stress [49]. Several studies have reported the protective effect of in vivo treatment with apocynin in experimental vascular injury models associated with ROS overproduction. Beswick et al. [52] and Ghosh et al. [53] showed that extended oral treatment of apocynin not only reduces superoxide generation in arteries isolated from deoxycorticosterone acetate salt- (DOCA-) induced hypertensive rats, but also reduces blood pressure associated with hypertension [52, 53]. Apocynin inhibits the generation of NADPH-derived superoxide and prevents the damaging interaction between NO and superoxide to form more potent ROS, thereby maintaining endothelial function despite the stimulus for arterial remodelling [54].

NADPH-oxidase is implicated in vascular remodelling and superoxide-stimulated cell proliferation in the neointima contributes to intimal hyperplasia in this collar model. Targeting NADPH-oxidase via adventitial drug delivery not only reduces superoxide generation, but also normalises endothelial cell function [54]. Targeting the primary source of NADPH-oxidase-derived superoxide is an effective approach to prevent deleterious arterial remodelling, providing a rationale for designing more efficacious and selective inhibitors of vascular NADPH-oxidase as potential therapeutics for human vascular disease. Reactive oxygen species (ROS) are thought to play an important role in atherogenesis. It has been demonstrated that the Nox2 catalytic component of NADPH-oxidase is upregulated in a non-hyperlipidemic rabbit model of early stage arterial remodelling [50, 55].

Apocynin has shown promising application in animal studies of hypertension and other cardiovascular diseases. In the Dahl salt-sensitive rat, apocynin inhibits superoxide production in the renal medulla and decreases hypertension [56]. DOCA-induced increases in aortic and renal superoxide production and hypertension are also attenuated by apocynin treatment [52, 57]. In this model, apocynin inhibits flow-induced superoxide production in the thick ascending loop of Henle, which is thought to mediate inappropriate NaCl retention in salt-sensitive hypertension [58] and prevents endothelial dysfunction [53]. Apocynin also prevents and reverses increases in systolic blood pressure (SBP) in dexamethasone-induced hypertension [59]. Furthermore, apocynin might be effective in treating humoral forms of hypertension such as those induced by Ang II and aldosterone.

Apocynin decreased p22phox mRNA levels in aortic segments from aldosterone-salt male Sprague-Dawley (SD) rats and impeded p47phox subunit assembly within the membrane complex in human endothelial cells to inhibit the activity of NAD(P)H oxidase and its production of superoxide. Apocynin prevents and reverses adrenocorticotropic hormone- (ACTH-) induced hypertension [2, 49], indicating that NAD(P)H oxidase is a major enzymatic source of superoxide overproduction in rat model of both naturally occurring and synthetic hypertension. Apocynin prevented and reversed Dex-induced changes in SBP, suggesting that upregulation of superoxide production in Dex-hypertension is related to increased NAD(P)H oxidase activity [35].

Apocynin may have potential negative effects, causing a defect in bactericidal phagocytosis which can mimic a chronic granulomatous disease (CGD) by indirect inhibition of respiratory burst. Nevertheless, Wilkins et al. [60] claim that NADPH-oxidase is not required for LDL oxidation by human monocyte-derived macrophages because human monocyte-derived macrophages (HMDMs) from chronic granulomatous disease patients were able to oxidize LDL as suggested by increased lipoprotein uptake by mouse macrophages.

On the other hand, Aviram et al. [61] state that activation of NADPH-oxidase is essential for macrophage-mediated oxidation of LDL. However, HMDM from patients with CGD that were shown to lack active NADPH-oxidase, but to possess almost normal 15-1ipoxygenase activity, failed to oxidize LDL. The study shows that addition of apocynin to an incubation system completely blocked the release of superoxides to the medium, suggesting that LDL-induced macrophage release of superoxides under oxidative stress is indeed associated with lipoprotein stimulation of cellular NADPH-oxidase activity.

Although it can be questionable if apocynin may indeed have potential negative effect triggering a defect in bactericidal phagocytosis which can mimic CGD, reversible character of action of apocynin and ambiguity of available data display that this problem still needs investigation.

## 6. APPLICATION: COX-2 AND CARTILAGE

Hougee et al. [5] confirmed two important features of apocynin in vivo: (1) oral administration of apocynin can partially reverse the inflammation-induced inhibition of cartilage proteoglycan synthesis, and (2) oral administration of apocynin has COX inhibitory effects similar to the nonsteroidal anti-inflammatory drug (NSAID) ibuprofen. Therefore, apocynin might be of potential use during the treatment of chronic inflammatory joint diseases like osteoarthritis or rheumatoid arthritis [5, 16].

Apocynin has been reported to inhibit the superoxide generating enzyme NADPH-oxidase [12, 13] present in chondrocytes [62, 63]. Peroxynitrite is the highly reactive coupling product of superoxide and nitric oxide and has been suggested to play a role in the inflammation-mediated inhibition of cartilage proteoglycan synthesis [43]. By inhibiting either NO or superoxide production, the amount of concurrently generated peroxynitrite would be decreased. Moreover, another recent study showed that apocynin prevented COX-2 expression in stimulated human monocyte [8]. The mechanism of action involved the inhibition of the NADPH-oxidase-dependent superoxide production, the reduction of the intracellular GSH/GSSG ratio, and the prevention of the activation of the nuclear transcription factor NF-*κ*B, which is an important mediator of inflammation [5, 64].

However, the recent finding that apocynin is capable of preventing COX-2 expression might provide an additional explanation for the anti-inflammatory effects of apocynin that have been observed in vivo. These in vivo effects of apocynin include the reduction of arthritis incidence [11], decreased joint swelling in collagen-induced arthritis in mice [27, 65], as well as the reduction of ulcerative skin lesions in inflamed skin in rats [66]. Apocynin also prevented airway hyperresponsiveness during allergic reactions in mice [67] and reduced airway hyperreactivity to metacholine when inhaled by humans suffering from mild atopic asthma [9]. The COX-2 enzyme and its major metabolite prostaglandin E_2_ (PGE_2_) play an important role during inflammatory diseases. For example, in osteoarthritis and rheumatoid arthritis, COX inhibitors such as NSAIDs are used to treat joint swelling and pain [68–70]. By inhibiting the formation of superoxide with apocynin, and hence reducing the amount of peroxynitrite, the inflammation-mediated reduction of proteoglycan synthesis might be restored. In vitro, this has been demonstrated in human cartilage [5, 6, 11].

## 7. OBJECTS

A serious potential problem comes from studies showing that apocynin may actually increase oxidative stress under some conditions. Glutathione expression is decreased in response to apocynin treatment in alveolar epithelial cells [16, 71] and apocynin activation by myeloperoxidase produces an apocynin-free radical that is able to oxidize glutathione [16]. Glial cells treated with apocynin show a dose-dependent increase in oxidative stress markers, including increases in oxidized glutathione. Unfortunately, apocynin must be administered at high doses for effectiveness [71].

Apocynin evoked, in a significant way, an increase of H_2_O_2_ concentration and a decrease of the intracellular glutathione/glutathione disulfide ratio, accompanied by augmented efflux of glutathione and glutathione disulfide. Apocynin induced the activation of both pentose phosphate pathway (PPP) and tricarboxylic acid cycle, which was blocked when the cells were incubated with glutathione together with apocynin [36, 47, 72]. The cell incubation with glutathione prevented also the apocynin-induced increase of malonyldialdehyde generation and lactate dehydrogenase leakage. Apocynin exerted an oxidant effect also in a cell-free system: indeed, in aqueous solution, it evoked a faster oxidation of the thiols glutathione and dithiothreitol, and elicited the generation of reactive oxygen species, mainly superoxide anions. This suggests that apocynin per se can induce an oxidative stress and exert a cytotoxic effect in different cell types, and that some effects of apocynin in experimental models in vitro and in vivo should be interpreted with caution [16, 47].

Also, according to Riganti et al. [73] apocynin is able to increase the H_2_O_2_ level in resting monocyte-like cells (i.e., the N11 glial cell line) and can induce, under longer times of exposure, an oxidative damage and a cytotoxic effect. Therefore, it is suggested that apocynin is per se an inducer of ROS production, independent of the cell type. It is conceivable that, when NADPH-oxidase is maximally activated, the inhibition of the respiratory burst is the prevailing effect of the drug, but in absence of a stimulation of NADPH-oxidase, the oxidative effect of apocynin itself could predominate. Additionally, Riganti et al. hypothesize that since sulfhydryl groups are important for the function of the leukocyte NADPH-oxidase, the oxidant effect of apocynin could participate to the mechanism of enzyme inhibition [71, 73].

Apocynin induces activation of both PPP and tricarboxylic acid cycle, which is subsequent to the oxidative stress, because the presence of GSH in the medium together with apocynin actually blocks the activation of both metabolic pathways. Riganti et al. [71] hypothesized that apocynin is able to increase the H_2_O_2_ level in resting monocyte-like cells (i.e., the N11 glial cell line) and can induce under longer times of exposure an oxidative damage and a cytotoxic effect. Therefore, it is suggested that apocynin is an inducer of ROS production, independent of the cell type. It is conceivable that when NADPH-oxidase is maximally activated, the inhibition of the respiratory burst is the prevailing effect of the drug, as a wide body of literature has already shown, but in absence of a stimulation of NADPH-oxidase, the oxidative effect of apocynin itself could predominate. It may be also hypothesized that since sulfhydryl groups are important for the function of the leukocyte NADPH-oxidase [13, 16], the oxidant effect of apocynin could participate to the mechanism of enzyme inhibition. It has been already reported that apocynin may exert other effects beside its ability to inhibit NADPH-oxidase: for instance, it inhibits cytochrome P450 activity in endothelial cells [74], interferes with actin polymerization and cytoskeletal rearrangement in polymorphonuclear granulocytes [17], and modulates the arachidonic acid metabolism through a not-yet-clarified mechanism [55, 71].

## 8. SCAVENGER PROPERTIES

According to Heumüller et al. [75], apocynin predominantly acts as an antioxidant but not as an inhibitor of NADPH-oxidases in nonphagocytic cells in culture. It failed to block the O_2_^−^ production of Nox1, Nox2, or Nox4 when overexpressed in endothelial cells. Moreover, in vascular cells, a similar activation of apocynin, as seen in leukocytes, was not observed. Most importantly, however, apocynin turned out to be a scavenger of radicals and directly inhibited the ROS-induced signaling in vascular cells [54, 76].

Titova et al. [39] found that apocynin and its oxidation products do not react with GSH. However, this thiol compound was efficiently oxidized by the apocynin radical during the MPO-catalyzed oxidation. The strong inhibitor effect of apocynin on the production of hypochlorous acid by stimulated neutrophils might be the result of an additive effect of NADPH-oxidase inhibition and, to a lesser extent in less extension, due to competition with chloride for the catalytic active site of MPO. This is a further evidence of the importance of apocynin as an anti-inflammatory drug [10]. Titova et al. [39] verified that neither apocynin nor the dimer and trimer derivatives were able to conjugate with GSH, which is a common representative of thiol compounds. However, they obtained strong evidence that GSH is able to react with apocynin radical and/or its dimer radical, which are formed during MPO-catalyzed oxidation.

Stolk et al. [13] showed that apocynin did not scavenge superoxide anion generated by xanthine oxidase. They concluded, however, that apocynin scavenged hydrogen peroxide because of a dose-response inhibition of luminol-enhanced chemiluminescence generated by hydrogen peroxide. The protective effect of apocynin may not be limited to NADPH-oxidase inhibition. Apocynin has been shown to inhibit cytochrome P450 [11] and thromboxane synthase [55]. Interestingly, both cytochrome P450 [77] and thromboxane [78] have been implicated as mediators of ischemia-reperfusion lung injury.

## 9. APOCYNIN AND CANCER

Klees et al. [79] reported that while apocynin itself is not effective, its derivatives inhibit migration of the breast cancer cell line MDAMB-435 at subtoxic concentrations and the migration of nonmalignant MCF10A breast cells is unaffected. These compounds also cause a significant rearrangement of the actin cytoskeleton, cell rounding, and decreased levels of active Rac1 and its related G protein Cdc42. The possible link between apocynin and Rac1 inhibition suggests that apocynin may be a source for inhibitors of Rac1-mediated tumor cell migration. Klees et al. reports the application of an in vitro screening assay to identify apocynin-derived inhibitors of Rac1-based tumor cell migration. According to this study, apocynin, upon peroxidase-catalyzed metabolic activation, interferes with NADPH-oxidase and inhibits lymphocyte migration through a G-protein-regulated pathway without affecting adhesion. Reactive oxygen species generated by NADPH-oxidase also control actin structure [80]. Apocynin or its metabolites have also been shown to affect the migration of polymorphonuclear granulocytes, suggesting that its mechanism of action is conserved throughout cell types [4, 79]. Active Rac1 is necessary for the translocation of p47-phox and p67-phox though it does not mediate it directly. Rac1's role in NADPH-oxidase activation is not well understood, but it is able to bind p67-phox, and this binding may be what causes the final formation of the active NADPH-oxidase complex. When Rac1 is in its inactive form, there is a decreased level of O_2_^−^, signifying inactive NADPH-oxidase [8]. NADPH-oxidase has also been shown to associate with the actin cytoskeleton, implicating another mode, by which Rac1 may manage cytoskeletal structure [49, 79].

## 10. CONCLUSION

Taken together, there is increasing support to consider using apocynin as a therapeutic agent for treatment of inflammatory diseases. The data of many experiments show that NADPH-oxidase is an important contributor to elevated levels of ROS. The property of apocynin as an inhibitor of NADPH-oxidase and its dependence on MPO-catalyzed oxidation might be linked to the reaction of apocynin radical and/or its dimer radical with intracellular GSH or directly with essential thiols of the cytosolic factor p47phox and this fact might be a key to further experiments, exploring wider application of apocynin [16, 81, 82].

Summarizing all the applications of apocynin and taking into account its low toxicity, selectivity, and lack of known side effects, it can be concluded that apocynin deserves further attention and that studies to elucidate its mode of action may contribute to the development of safe and selective anti-inflammatory drugs which lack the often serious side effects of steroids.

## References

[1] Chlopicki S, Olszanecki R, Janiszewski M, Laurindo FRM, Panz T, Miedzobrodzki J (2004). Functional role of NADPH oxidase in activation of platelets. *Antioxidants & Redox Signaling*.

[2] Stolk J, Hiltermann TJ, Dijkman JH, Verhoeven AJ (1994). Characteristics of the inhibition of NADPH oxidase activation in neutrophils by apocynin, a methoxy-substituted catechol. *American Journal of Respiratory Cell and Molecular Biology*.

[3] Li J-M, Gall NP, Grieve DJ, Chen M, Shah AM (2002). Activation of NADPH oxidase during progression of cardiac hypertrophy to failure. *Hypertension*.

[4] Luchtefeld R, Luo R, Stine K, Alt ML, Chernovitz PA, Smith RE (2008). Dose formulation and analysis of diapocynin. *Journal of Agricultural and Food Chemistry*.

[5] Hougee S, Hartog A, Sanders A (2006). Oral administration of the NADPH-oxidase inhibitor apocynin partially restores diminished cartilage proteoglycan synthesis and reduces inflammation in mice. *European Journal of Pharmacology*.

[6] Lafeber FPJG, Beukelman CJ, van den Worm E (1999). Apocynin, a plant-derived, cartilage-saving drug, might be useful in the treatment of rheumatoid arthritis. *Rheumatology*.

[7] Zhang Y, Chan MMK, Andrews MC (2005). Apocynin but not allopurinol prevents and reverses adrenocorticotropic hormone-induced hypertension in the rat. *American Journal of Hypertension*.

[8] Barbieri SS, Cavalca V, Eligini S (2004). Apocynin prevents cyclooxygenase 2 expression in human monocytes through NADPH oxidase and glutathione redox-dependent mechanisms. *Free Radical Biology and Medicine*.

[9] Peters EA, Hiltermann JTN, Stolk J (2001). Effect of apocynin on ozone-induced airway hyperresponsiveness to methacholine in asthmatics. *Free Radical Biology and Medicine*.

[10] Johnson DK, Schillinger KJ, Kwait DM (2002). Inhibition of NADPH oxidase activation in endothelial cells by ortho-methoxy-substituted catechols. *Endothelium*.

[11] Smit HF, Kroes BH, van den Berg AJJ (2000). Immunomodulatory and anti-inflammatory activity of *Picrorhiza scrophulariiflora*. *Journal of Ethnopharmacology*.

[12] Simons JM, 't Hart BA, Ip Vai Ching TRAM, Van Dijk H, Labadie RP (1990). Metabolic activation of natural phenols into selective oxidative burst agonists by activated human neurophils. *Free Radical Biology and Medicine*.

[13] Stolk J, Rossie W, Dijkman JH (1994). Apocynin improves the efficacy of secretory leukocyte protease inhibitor in experimental emphysema. *American Journal of Respiratory and Critical Care Medicine*.

[14] van den Worm E, Beukelman CJ, van den Berg AJJ, Kroes BH, Labadie RP, Van Dijk H (2001). Effects of methoxylation of apocynin and analogs on the inhibition of reactive oxygen species production by stimulated human neutrophils. *European Journal of Pharmacology*.

[15] Clark RA, Volpp BD, Leidal KG, Nauseef WM (1990). Two cytosolic components of the human neutrophil respiratory burst oxidase translocate to the plasma membrane during cell activation. *Journal of Clinical Investigation*.

[16] Ximenes VF, Kanegae MPP, Rissato SR, Galhiane MS (2007). The oxidation of apocynin catalyzed by myeloperoxidase: proposal for NADPH oxidase inhibition. *Archives of Biochemistry and Biophysics*.

[17] Müller AA, Reiter SA, Heider KG, Wagner H (1999). Plant-derived acetophenones with antiasthmatic and anti-inflammatory properties: inhibitory effects on chemotaxis, right angle light scatter and actin polymerization of polymorphonuclear granulocytes. *Planta Medica*.

[18] Muijsers RBR, van den Worm E, Folkerts G (2000). Apocynin inhibits peroxynitrite formation by murine macrophages. *British Journal of Pharmacology*.

[19] Daly JW, Axelrod J, Witkop B (1960). Dynamic aspects of enzymatic O-methylation and -demethylation of catechols in vitro and in vivo. *The Journal of Biological Chemistry*.

[20] Gajewska ZG, Grzybowski J (1981). Analysis of an industrial smoke preparation. *Bromatologia i Chemia Toksykologiczna*.

[21] Dodd-o JM, Welsh LE, Salazar JD (2004). Effect of NADPH oxidase inhibition on cardiopulmonary bypass-induced lung injury. *American Journal of Physiology*.

[22] Pearse DB, Dodd-o JM (1999). Ischemia-reperfusion lung injury is prevented by apocynin, a novel inhibitor of leukocyte NADPH oxidase. *Chest*.

[23] Al-Mehdi AB, Zhao G, Dodia C (1998). Endothelial NADPH oxidase as the source of oxidants in lungs exposed to ischemia or high K^+^. *Circulation Research*.

[24] Minamiya Y, Tozawa K, Kitamura M, Saito S, Ogawa J-I (1998). Platelet-activating factor mediates intercellular adhesion molecule-1-dependent radical production in the nonhypoxic ischemia rat lung. *American Journal of Respiratory Cell and Molecular Biology*.

[25] Dodd-o JM, Pearse DB (2000). Effect of the NADPH oxidase inhibitor apocynin on ischemia-reperfusion lung injury. *American Journal of Physiology*.

[26] Pearse DB, Wagner EM, Sylvester JT (1993). Edema clearance in isolated sheep lungs. *Journal of Applied Physiology*.

[27] 't Hart BA, Simons JM, Knaan-Shanzer S, Bakker NPM, Labadie RP (1990). Antiarthritic activity of the newly developed neutrophil oxidative burst antagonist apocynin. *Free Radical Biology and Medicine*.

[28] Salmon M, Koto H, Lynch OT (1998). Proliferation of airway epithelium after ozone exposure: effect of apocynin and dexamethasone. *American Journal of Respiratory and Critical Care Medicine*.

[29] Lapperre TS, Jimenez LA, Antonicelli F (1999). Apocynin increases glutathione synthesis and activates AP-1 in alveolar epithelial cells. *FEBS Letters*.

[30] Nishikawa M, Kudo M, Kakemizu N, Ikeda H, Okubo T (1996). Role of superoxide anions in airway hyperresponsiveness induced by cigarette smoke in conscious guinea pigs. *Lung*.

[31] Hamid Q, Springall DR, Riveros-Moreno V (1993). Induction of nitric oxide synthase in asthma. *The Lancet*.

[32] Sadeghi-Hashjin G, Folkerts G, Henricks PAJ, Muijsers RBR, Nijkamp FP (1998). Peroxynitrite in airway diseases. *Clinical & Experimental Allergy*.

[33] Muijsers RBR, Folkerts G, Henricks PAJ, Sadeghi-Hashjin G, Nijkamp FP (1997). Peroxynitrite: a two-faced metabolite of nitric oxide. *Life Sciences*.

[34] Sadeghi-Hashjin G, Folkerts G, Henricks PAJ (1996). Peroxynitrite induces airway hyperresponsiveness in guinea pigs in vitro and in vivo. *American Journal of Respiratory and Critical Care Medicine*.

[35] Tang LL, Ye K, Yang XF, Zheng JS (2007). Apocynin attenuates cerebral infarction after transient focal ischaemia in rats. *Journal of International Medical Research*.

[36] Wang Q, Tompkins KD, Simonyi A, Korthuis RJ, Sun AY, Sun GY (2006). Apocynin protects against global cerebral ischemia-reperfusion-induced oxidative stress and injury in the gerbil hippocampus. *Brain Research*.

[37] Zheng JS, Zhan RY, Zheng SS, Zhou YQ, Tong Y, Wan S (2005). Inhibition of NADPH oxidase attenuates vasospasm after experimental subarachnoid hemorrhage in rats. *Stroke*.

[38] Lo W, Bravo T, Jadhav V, Titova E, Zhang JH, Tang J (2007). NADPH oxidase inhibition improves neurological outcomes in surgically-induced brain injury. *Neuroscience Letters*.

[39] Titova E, Ostrowski RP, Sowers LC, Zhang JH, Tang J (2007). Effects of apocynin and ethanol on intracerebral haemorrhage-induced brain injury in rats. *Clinical and Experimental Pharmacology and Physiology*.

[40] Tang XN, Cairns B, Cairns N, Yenari MA (2008). Apocynin improves outcome in experimental stroke with a narrow dose range. *Neuroscience*.

[41] Crawford DW, Blankenhorn DH (1991). Arterial wall oxygenation, oxyradicals, and atherosclerosis. *Atherosclerosis*.

[42] Hessler JR, Morel DW, Lewis LJ, Chisolm GM (1983). Lipoprotein oxidation and lipoprotein-induced cytotoxicity. *Arteriosclerosis*.

[43] Holland JA, Meyer JW, Chang M-M, O'Donnell RW, Johnson DK, Ziegler LM (1998). Thrombin stimulated reactive oxygen species production in cultured human endothelial cells. *Endothelium*.

[44] Holland JA, Meyer JW, Schmitt ME (1997). Low-density lipoprotein stimulated peroxide production and endocytosis in cultured human endothelial cells: mechanisms of action. *Endothelium*.

[45] Holland JA Prevention of atherosclerosis using NADPH oxidase inhibitors.

[46] Meyer JW, Holland JA, Ziegler LM, Chang M-M, Beebe G, Schmitt ME (1999). Identification of a functional leukocyte-type NADPH oxidase in human endothelial cells: a potential atherogenic source of reactive oxygen species. *Endothelium*.

[47] Meyer JW, Schmitt ME (2000). A central role for the endothelial NADPH oxidase in atherosclerosis. *FEBS Letters*.

[48] Diatchuk V, Lotan O, Koshkin V, Wikstroem P, Pick E (1997). Inhibition of NADPH oxidase activation by 4-(2-aminoethyl)-benzenesulfonyl fluoride and related compounds. *The Journal of Biological Chemistry*.

[49] Hamilton CA, Brosnan MJ, Al-Benna S, Berg G, Dominiczak AF (2002). NAD(P)H oxidase inhibition improves endothelial function in rat and human blood vessels. *Hypertension*.

[50] Paravicini TM, Gulluyan LM, Dusting GJ, Drummond GR (2002). Increased NADPH oxidase activity, gp91phox expression, and endothelium-dependent vasorelaxation during neointima formation in rabbits. *Circulation Research*.

[51] Dusting GJ, Curcio A, Harris PJ, Lima B, Zambetis M, Martin JF (1990). Supersensitivity to vasoconstrictor action of serotonin precedes the development of atheroma-like lesions in the rabbit. *Journal of Cardiovascular Pharmacology*.

[52] Beswick RA, Dorrance AM, Leite R, Webb RC (2001). NADH/NADPH oxidase and enhanced superoxide production in the mineralocorticoid hypertensive rat. *Hypertension*.

[53] Ghosh M, Wang HD, McNeill JR (2004). Role of oxidative stress and nitric oxide in regulation of spontaneous tone in aorta of DOCA-salt hypertensive rats. *British Journal of Pharmacology*.

[54] Chan EC, Datla SR, Dilley R, Hickey H, Drummond GR, Dusting GJ (2007). Adventitial application of the NADPH oxidase inhibitor apocynin in vivo reduces neointima formation and endothelial dysfunction in rabbits. *Cardiovascular Research*.

[55] Engels F, Renirie BF, 't Hart BA, Labadie RP, Nijkamp FP (1992). Effects of apocynin, a drug isolated from the roots of *Picrorhiza kurroa*, on arachidonic acid metabolism. *FEBS Letters*.

[56] Taylor NE, Glocka P, Liang M, Cowley AW (2006). NADPH oxidase in the renal medulla causes oxidative stress and contributes to salt-sensitive hypertension in Dahl S rats. *Hypertension*.

[57] Jin L, Beswick RA, Yamamoto T (2006). Increased reactive oxygen species contributes to kidney injury in mineralocorticoid hypertensive rats. *Journal of Physiology and Pharmacology*.

[58] Hong NJ, Garvin JL (2007). Flow increases superoxide production by NADPH oxidase via activation of Na-K-2Cl cotransport and mechanical stress in thick ascending limbs. *American Journal of Physiology*.

[59] Hu L, Zhang Y, Lim PS (2006). Apocynin but not L-arginine prevents and reverses dexamethasone-induced hypertension in the rat. *American Journal of Hypertension*.

[60] Wilkins GM, Segal AW, Leake DS (1994). NADPH oxidase is not essential for low density lipoprotein oxidation by human monocyte-derived macrophages. *Biochemical and Biophysical Research Communications*.

[61] Aviram M, Rosenblat M, Etzioni A, Levy R (1996). Activation of NADPH oxidase is required for macrophage-mediated oxidation of low-density lipoprotein. *Metabolism*.

[62] Hiran TS, Moulton PJ, Hancock JT (1997). Detection of superoxide and NAPDH oxidase in porcine articular chondrocytes. *Free Radical Biology and Medicine*.

[63] Moulton PJ, Goldring MB, Hancock JT (1998). NADPH oxidase of chondrocytes contains an isoform of the gp91phox subunit. *Biochemical Journal*.

[64] Barnes PJ, Karin M (1997). Nuclear factor-*κ*B—a pivotal transcription factor in chronic inflammatory diseases. *The New England Journal of Medicine*.

[65] 't Hart BA, Bakker NP, Labadie RP, Simons JM (1991). The newly developed neutrophil oxidative burst antagonist apocynin inhibits joint-swelling in rat collagen arthritis. *Agents and Actions Supplements*.

[66] 't Hart BA, Elferink JGR, Nibbering PH (1992). Effect of apocynin on the induction of ulcerative lesions in rat skin injected with tubercle bacteria. *International Journal of Immunopharmacology*.

[67] Muijsers RBR, van Ark I, Folkerts G (2001). Apocynin and 1400 W prevents airway hyperresponsiveness during allergic reactions in mice. *British Journal of Pharmacology*.

[68] Bradley JD, Brandt KD, Katz BP, Kalasinski LA, Ryan SI (1992). Treatment of knee osteoarthritis: relationship of clinical features of joint inflammation to the response to a nonsteroidal antiinflammatory drug or pure analgesic. *Journal of Rheumatology*.

[69] Collantes E, Curtis SP, Lee KW (2002). A multinational randomized, controlled, clinical trial of etoricoxib inthetreatment of rheumatoid arthritis [ISRCTN25142273]. *BMC Family Practice*.

[70] FitzGerald GA, Patrono C (2001). The coxibs, selective inhibitors of cyclooxygenase-2. *The New England Journal of Medicine*.

[71] Riganti C, Costamagna C, Bosia A, Ghigo D (2006). The NADPH oxidase inhibitor apocynin (acetovanillone) induces oxidative stress. *Toxicology and Applied Pharmacology*.

[72] Kudo M, Nishikawa M, Ikeda H, Okubo T (1996). Involvement of superoxide anions in ozone-induced airway hyperresponsiveness in unanesthetized guinea pigs. *Environmental Toxicology and Pharmacology*.

[73] Riganti C, Costamagna C, Doublier S (2008). The NADPH oxidase inhibitor apocynin induces nitric oxide synthesis via oxidative stress. *Toxicology and Applied Pharmacology*.

[74] Pietersma A, de Jong N, de Wit LE, Kraak-Slee RG, Koster JF, Sluiter W (1998). Evidence against the involvement of multiple radical generating sites in the expression of the vascular cell adhesion molecule-1. *Free Radical Research*.

[75] Heumüller S, Wind S, Barbosa-Sicard E (2008). Apocynin is not an inhibitor of vascular NADPH oxidases but an antioxidant. *Hypertension*.

[76] Akard LP, English D, Gabig TG (1988). Rapid deactivation of NADPH oxidase in neutrophils: continuous replacement by newly activated enzyme sustains the respiratory burst. *Blood*.

[77] Bysani GK, Kennedy TP, Ky N, Rao NV, Blaze CA, Hoidal JR (1990). Role of cytochrome P-_450_ in reperfusion injury of the rabbit lung. *The Journal of Clinical Investigation*.

[78] Ljungman AG, Grum CM, Deeb GM, Bolling SF, Morganroth ML (1991). Inhibition of cyclooxygenase metabolite production attenuates ischemia-reperfusion lung injury. *American Review of Respiratory Disease*.

[79] Klees RF, De Marco PC, Salasznyk RM (2006). Apocynin derivatives interrupt intracellular signaling resulting in decreased migration in breast cancer cells. *Journal of Biomedicine and Biotechnology*.

[80] Matheny HE, Deem TL, Cook-Mills JM (2000). Lymphocyte migration through monolayers of endothelial cell lines involves VCAM-1 signaling via endothelial cell NADPH oxidase. *The Journal of Immunology*.

[81] Vejražka M, Míček R, Štípek S (2005). Apocynin inhibits NADPH oxidase in phagocytes but stimulates ROS production in non-phagocytic cells. *Biochimica et Biophysica Acta*.

[82] Niessen HWM, Kuijpers TW, Roos D, Verhoeven AJ (1991). Release of azurophil granule contents in fMLP-stimulated neutrophils requires two activation signals, one of which is a rise in cytosolic free Ca^2+^. *Cellular Signalling*.

